# The Hopf whole-brain model and its linear approximation

**DOI:** 10.1038/s41598-024-53105-0

**Published:** 2024-01-31

**Authors:** Adrián Ponce-Alvarez, Gustavo Deco

**Affiliations:** 1 Departament de Matemàtiques, Universitat Politècnica de Catalunya, 08028 Barcelona, Spain; 2 Center for Brain and Cognition, Computational Neuroscience Group, Department of Information and Communication Technologies, Universitat Pompeu Fabra, 08005 Barcelona, Spain; 3 Institució Catalana de la Recerca i Estudis Avançats (ICREA), 08010 Barcelona, Spain

**Keywords:** Computational neuroscience, Neural circuits

## Abstract

Whole-brain models have proven to be useful to understand the emergence of collective activity among neural populations or brain regions. These models combine connectivity matrices, or *connectomes*, with local node dynamics, noise, and, eventually, transmission delays. Multiple choices for the local dynamics have been proposed. Among them, nonlinear oscillators corresponding to a supercritical Hopf bifurcation have been used to link brain connectivity and collective phase and amplitude dynamics in different brain states. Here, we studied the linear fluctuations of this model to estimate its stationary statistics, i.e., the instantaneous and lagged covariances and the power spectral densities. This linear approximation—that holds in the case of heterogeneous parameters and time-delays—allows analytical estimation of the statistics and it can be used for fast parameter explorations to study changes in brain state, changes in brain activity due to alterations in structural connectivity, and modulations of parameter due to non-equilibrium dynamics.

## Introduction

Whole-brain models are coupled stochastic dynamical systems in which nodes (i.e., brain regions) interact through couplings that represent anatomical connections estimated using diffusion imaging^1^, fiber tracing techniques^2^, or generative rules—such as the exponential distance rule^3^. Whole-brain models have proven to be useful to understand the emergence of correlations between neural populations or brain regions (or functional connectivity), as well as their spectral properties, in different brain states. In general, the ingredients of these models are a connectivity matrix between nodes, local node dynamics, noise, and, eventually, time-delays. Multiple choices for the local dynamics have been used depending on the studied behavior (e.g., network correlations, synchrony, metastability, etc.) and the data to be modelled (e.g., fMRI or M/EEG). Local node dynamics have been previously modelled using spiking networks^4^, conductance-based dynamics^1^, neural population dynamics^5^, neural mass models^6^, excitable systems^7^, phase oscillators^8–10^, and nonlinear oscillators^11^. In the present study, we examined the behavior of a network of nonlinear oscillators corresponding to a normal form of a supercritical Hopf bifurcation. This network model, first introduced by Matthews and Strogatz^12^ to study collective behavior, is known as the Stuart-Landau model. It is a canonical model to study systems of coupled oscillators for which both the phase and the amplitude interact. The Stuart-Landau network has been used in diverse applications, from the study of coupled lasers^13^ to neural networks^14^. In the context of neuroscience, this model is often referred as the Hopf model. In this model, as nonlinearities increase, isolated nodes transit through two qualitatively different dynamics: from damped oscillations to self-sustained oscillations.

The Hopf model has been used to study the link between brain structure and dynamics in resting-state conditions^15^ and in different brain states, such as sleep^16^, low-level states of consciousness^17–21^, and psychedelic states^22^. Moreover, the Stuart-Landau model has been used to study the emergence of remote synchronization in human cerebral cortex^23^. Theoretical works have revealed sophisticate nonlinear emergent phenomena in the Stuart-Landau network such as oscillation and amplitude death^24,25^. Nevertheless, comparison of whole-brain models with resting-state neuroimaging data showed that the network operates in the simpler noisy-oscillation regime, suggesting that nonlinearities are small^15,26^. As we showed below, this case allows to strongly simplify the model to estimate the network statistics. This is important because, the Hopf model being a system of coupled stochastic differential equations, estimation of the network statistics (e.g., variances and covariances) requires extensive numerical simulations^27^, making often unpractical the exploration of a large part of the model’s parameter space.

Here, we reviewed the Hopf model and derived network statistics using its linear approximation. The linearization allows analytical estimation of the statistics and can be used for fast parameter explorations without the need of extensive simulations. In order to facilitate future research, we have made the Matlab codes freely available online, allowing to perform the calculations for any connectome and for a large space of model parameters.

## Results

### Local dynamics

The dynamics of an isolated node are described by the following complex-valued equation, representing the normal form of a supercritical Hopf bifurcation:

1 $$\frac{dz}{dt}=\dot{z}=\left(a+{\text{i}}\omega \right)z-\kappa {\left|z\right|}^{2}z+\eta ,$$

where $z=x+{\text{i}}y$, with $x$ and $y$ being the real and imaginary parts of the state variable $z$ (which has arbitrary units), respectively, and ${\text{i}}$ the imaginary unit; $\left|z\right|$ is the module of $z$, i.e., ${\left|z\right|}^{2}={x}^{2}+{y}^{2}$; $\omega =2\pi \upsilon$ is the intrinsic angular frequency (in rad.s^-1^), where $\upsilon$ is the intrinsic frequency in Hz; the parameter $a$ is called the node’s bifurcation parameter (in s^-1^); finally, $\kappa$ is a dimensional parameter, equal to $\kappa$ = 1 s^−1^, that we dropped in the remaining of the article. Additive white noise is represented by $\eta$, i.e., $\langle \eta (t)\rangle =0$ and $\langle \eta (t)\eta ({t}^{\prime})\rangle ={\sigma }^{2}\delta (t-{t}^{\prime})$, where $\sigma$ is the noise amplitude (in s^−1/2^) and the angular brackets $\langle .\rangle$ denote the average over stochastic realizations. Note that we use the common, but loosely, notation for stochastic differential equations. Rigorously, Eq. (1) writes: $dz=\left[\left(a+{\text{i}}\omega \right)z-{\left|z\right|}^{2}z\right]dt+dW$, where $W$ represents a Wiener process (Brownian motion) for which a time-derivative is not defined and $\langle dW(t)dW(t)\rangle ={\sigma }^{2}dt$.

One can write Eq. (1) as a function of the real and imaginary parts:

2 $$\frac{dx}{dt}=\left(a-{x}^{2}-{y}^{2}\right)x-\omega y+{\eta }_{x},$$

3 $$\frac{dy}{dt}=\left(a-{x}^{2}-{y}^{2}\right)y+\omega x+{\eta }_{y},$$

where ${\eta }_{x}$ and ${\eta }_{y}$ are uncorrelated white noises added to the real and imaginary parts, respectively.

The variable $z$ can also be written in polar coordinates, i.e., $z=r{e}^{{\text{i}}\theta }$, where $r=\left|z\right|={\left({x}^{2}+{y}^{2}\right)}^{1/2}$ is the module of $z$ and $\theta ={\text{arctan}}\left(y/x\right)$ is its phase. Note that $r\ge 0$. In polar coordinates, we have $r\dot{r}=x\dot{x}+y\dot{y}$ and ${r}^{2}\dot{\theta }=x\dot{y}-y\dot{x}$. Thus, in absence of noise, Eq. (1) becomes:

4 $$\frac{d\theta }{dt}=\omega ,$$

5 $$\frac{dr}{dt}=ar-{r}^{3}.$$

**Figure 1:**
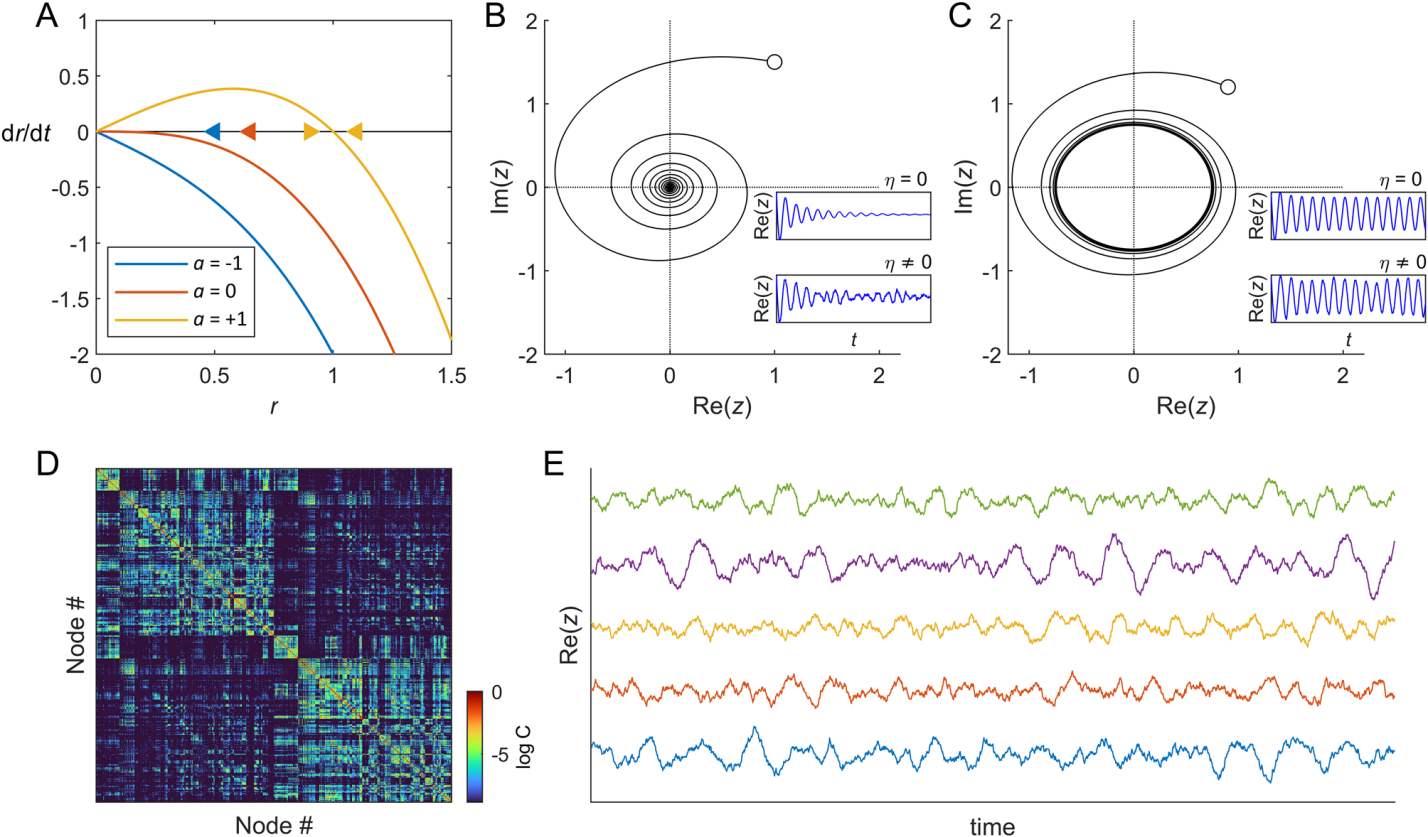
Hopf model: single-node and network dynamics. (**A**) The fixed points of a Hopf node have modules which are the roots of $\dot{r}=ar-{r}^{3}$. For $a<0$, the solution $r=0$ is stable since deviations from $r=0$ are attenuated (i.e., $\dot{r}<0$). On the contrary, if $a>0$, $r=0$ is unstable as fluctuations around it are amplified (i.e., $\dot{r}>0$). In this latter case a new fixed point appears given by $r={a}^{1/2}$, which is stable since fluctuations around it, $r={a}^{1/2}+\delta r$, are increased if $\delta r<0$, but decreased if $\delta r>0$. The arrows indicate the direction of flow and are given by the sign of $\dot{r}$. (**B**) Single-node dynamics for $a<0$. The system relaxes with damped oscillations from the initial condition (white circle) to the origin of the complex plane. Insets: *top*: in the absence of noise ($\eta =0$) the oscillations die out; *bottom*: in the presence of noise ($\eta \ne 0$) the oscillations are noise-driven. (**C**) Single-node dynamics for $a>0$. The system produces self-sustained oscillations. Insets: *top*, deterministic system; *bottom*, stochastic system. (**D**) Network model. The whole-brain network is composed of $N$ Hopf nodes interconnected through anatomical connections. Here, we used dMRI connectivity from the Human Connectome Project (HCP), in a parcellation with $N$ = 1000 nodes. (**E**) Example dynamics for five nodes of the network. Parameters: ${a}_{j}=-0.5$ (homogeneous); $g=1$; ${\omega }_{j}=10$ rad.s^-1^; $\sigma =0.3$.

Equation (4) indicates that the phase evolves independently of $r$ as $\theta \left(t\right)=\omega t+\varphi$, where $\varphi$ is a constant phase. Clearly, a fixed point of Eq. (5) is $r=0$ for which $\frac{dr}{dt}=0$. The stability of the fixed point $r=0$ depends on the parameter $a$, since deviations from $r=0$ grow (i.e., $\frac{dr}{dt}>0$) if $ar-{r}^{3}>0$ and decrease (i.e., $\frac{dr}{dt}< 0$) if $ar-{r}^{3}< 0$ (Fig. 1A). For $a<0$, the solution $r=0$ is stable as fluctuations around this point are attenuated. The eigenvalues of the system (2)–(3) are complex conjugates and equal to $\lambda =a\pm {\text{i}}\omega$. For $a<0$, both eigenvalues have negative real part, indicating that the system relaxes to $z=0$ with damped oscillations (see Fig. 1B), i.e., a *spiral* or *focus* solution. Note that, in this regime, addition of noise induces oscillations of the system. On the contrary, if $a>0$, $r=0$ is unstable as fluctuations around it are amplified (Fig. 1A). In this latter case, a new fixed point appears given by $r={a}^{1/2}$, which is stable since fluctuations around it, $r={a}^{1/2}+\delta r$, are increased if $\delta r<0$, but decreased if $\delta r>0$. This solution is called a *limit-cycle* for which the system produces self-sustained oscillations with a constant amplitude and a constant angular frequency $\omega$ (see Fig. 1C).

In studies of whole-brain models, the brain signals (e.g. fMRI or MEG) are modelled by the real part of the state variables, i.e., $x={\text{Re}}\left(z\right)$.

### Network model

The whole-brain dynamics are obtained by coupling the local dynamics of $N$ Hopf nodes interconnected through a given coupling matrix ${\varvec{C}}$ representing anatomical connections (Fig. 1C). In this study, to illustrate the method, we used a publicly available human diffusion MRI (dMRI) connectome from the Human Connectome Project (HCP) as the coupling matrix (${\varvec{C}}$). The state variables of the network are given by the system of stochastic coupled nonlinear differential equations:

6 $$\frac{d{z}_{j}}{dt}=\left({a}_{j}+{\text{i}}{\omega }_{j}\right){z}_{j}-{\left|{z}_{j}\right|}^{2}{z}_{j}+g\sum_{k=1}^{N}{C}_{jk}\left({z}_{k}-{z}_{j}\right)+{\eta }_{j},$$

where $g$ (in s^−1^) represents a global scaling of the connectivity ${\varvec{C}}$ and ${\eta }_{j}$ is uncorrelated white noise, i.e., $\langle {\eta }_{j}(t)\rangle =0$ and $\langle {\eta }_{j}{\left(t\right)\eta }_{k}({t}^{\prime})\rangle ={\sigma }^{2}\delta (t-{t}^{\prime}){\delta }_{jk}$. Two versions of this model have been studied previously: the homogenous case for which the local bifurcation parameter is constant across nodes (i.e., ${a}_{j}=a$)^15,16, 28^ and the heterogeneous case for which nodes can have different local bifurcation parameters ${a}_{j}$ estimated from the data^15,20^. In both cases, ${\omega }_{j}$ are estimated from the peak frequency of the data.

This model can be interpreted as an extension of the Kuramoto model to the case in which both the phase and the amplitude of the oscillators are allowed to vary and interact. In particular, the choice of the coupling function $\left({z}_{k}-{z}_{j}\right)$ promotes phase synchronization between coupled nodes. This can be seen by writing the deterministic system in polar coordinates:

7 $$\frac{d{\theta }_{j}}{dt}={\omega }_{j}+g\sum_{k=1}^{N}{C}_{jk}\frac{{r}_{k}}{{r}_{j}}{\text{sin}}\left({\theta }_{k}-{\theta }_{j}\right),$$

8 $$\frac{d{r}_{j}}{dt}=\left({a}_{j}-g\sum_{k=1}^{N}{C}_{jk}-{r}_{j}^{2}\right){r}_{j}+g\sum_{k=1}^{N}{C}_{jk}{r}_{k}{\text{cos}}\left({\theta }_{k}-{\theta }_{j}\right).$$

Equation (7) represents a version of the Kuramoto model of phase oscillators for which couplings are modulated by the ratio of the amplitudes. The term ${\text{sin}}\left({\theta }_{k}-{\theta }_{j}\right)$ favors synchronization of nodes $j$ and $k$, since an oscillator lagging behind another one (${\theta }_{k}-{\theta }_{j}>0$) is sped up (a positive term ${\text{sin}}\left({\theta }_{k}-{\theta }_{j}\right)$ is added), whereas an oscillator leading another (${\theta }_{k}-{\theta }_{j}<0$) is slowed down (a negative term ${\text{sin}}\left({\theta }_{k}-{\theta }_{j}\right)$ is added). In the case where the oscillations of the nodes are self-sustained (limit-cycles) and the couplings are weak, amplitude fluctuations are little compared to phase changes, and the system can be approximated by a Kuramoto model of phase oscillators interacting through couplings equal to ${C}_{jk}\sqrt{\frac{{a}_{k}}{{a}_{j}}}$. In this study, however, we concentrated on the case of noisy oscillations (i.e., when nodes do not produce self-sustained oscillations).

Note also that the coupling function can have a stabilizing effect, since Eq. (6) without noise can be written as: $\dot{{z}_{j}}=\left({a}_{j}-g{S}_{j}+{\text{i}}{\omega }_{j}\right){z}_{j}-{\left|{z}_{j}\right|}^{2}{z}_{j}+g\sum_{k=1}^{N}{C}_{jk}$, where ${S}_{j}=\sum_{k=1}^{N}{C}_{jk}$ is the strength of node $j$. In the case of ${S}_{j}>0$, which is true in particular for positive connections ${C}_{jk}$, the term $-g{S}_{j}<0$ contributes to the stability of the network.

### Linear approximation

Estimating the network statistics (e.g., the covariance matrix) of the system given by Eq. (6) requires long stochastic simulations, impeding the exploration of different model parameters. However, in the case of weak noise and small non-linearities, one can estimate the statistics of the whole-brain network using a linear approximation that we describe in this section.

In the following, we use bold symbols to indicate column vectors and matrices. The dynamical system can be re-written in vector form as:

9 $$\frac{{d{\varvec{z}}}}{dt} = \left( {{\varvec{a}} - g{\varvec{S}} + {\text{i}}{\varvec{\omega}}} \right) \odot {\varvec{z}} - \left( {{\varvec{z}} \odot \overline{\user2{z}}} \right){\varvec{z}} + g{\varvec{Cz}} + {\varvec{\eta}},$$

where ${\varvec{z}}={\left[{z}_{1}, \dots ,{z}_{N}\right]}^{T}$, $\overline{{\varvec{z}} }$ is the complex conjugate of ${\varvec{z}}$, ${\varvec{a}}={\left[{a}_{1}, \dots ,{a}_{N}\right]}^{T}$, ${\varvec{\omega}}={\left[{\omega }_{1}, \dots ,{\omega }_{N}\right]}^{T}$, ${\varvec{S}}={\left[{S}_{1}, \dots ,{S}_{N}\right]}^{T}$ is the vector containing the strength of each node, i.e., ${S}_{i}={\sum }_{j}{C}_{ij}$, and ${\varvec{\eta}}={\left[{\eta }_{1}, \dots ,{\eta }_{N}\right]}^{T}$ represents a vector of uncorrelated noise. The symbol $\odot$ is the Hadamard element-wise product, i.e., ${\mathbf{u}} \odot {\mathbf{v}} = \left[ {{\text{u}}_{1} {\text{v}}_{1} , \ldots ,{\text{u}}_{N} {\text{v}}_{N} } \right]^{T}$. The superscript $T$ denotes the transpose operator.

**Figure 2:**
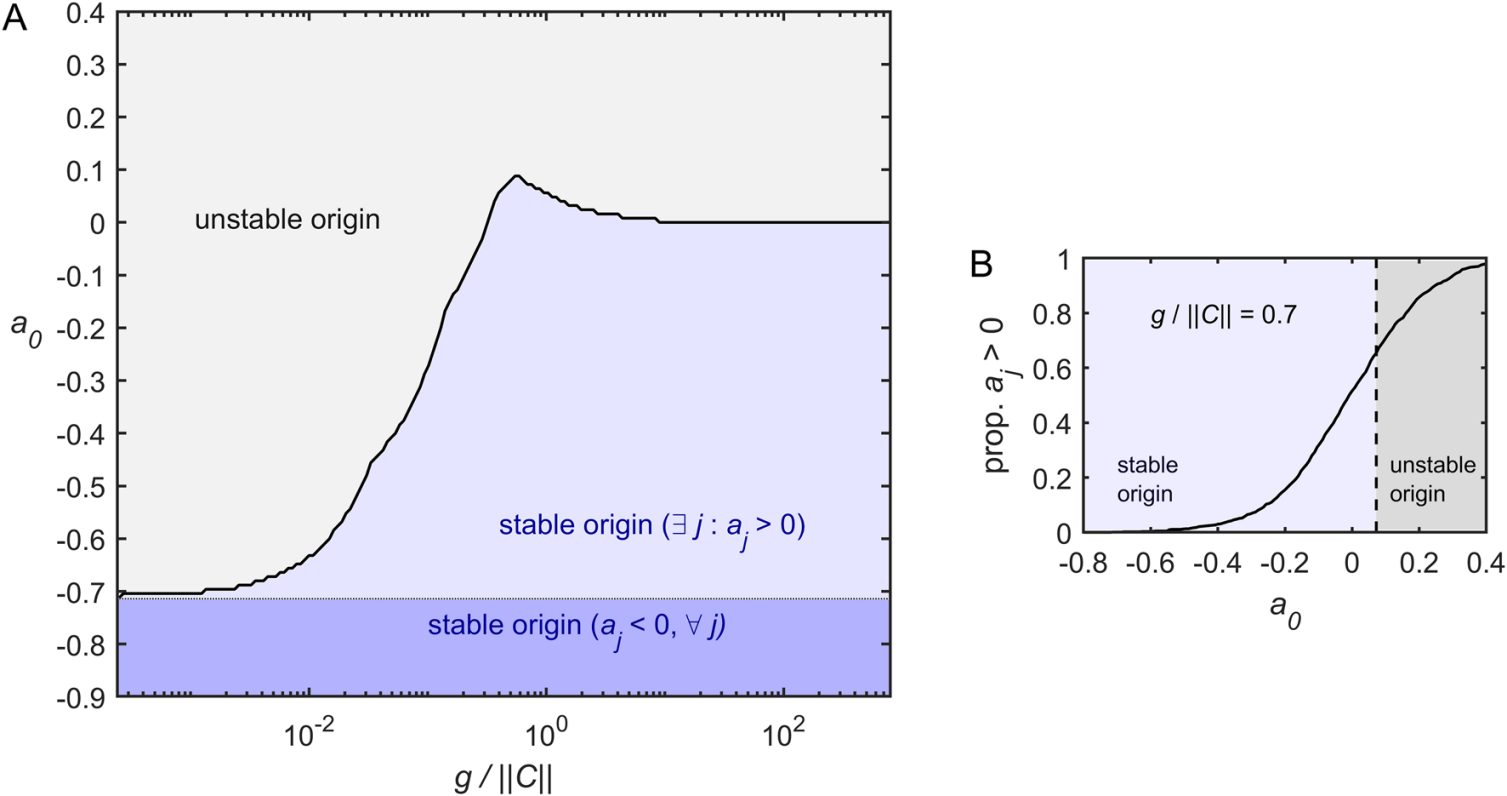
Linear stability of the origin. (**A**) We considered the heterogenous model for which the parameters ${\varvec{a}}$ and ${\varvec{\omega}}$ were drawn from normal distributions $\mathcal{N}\left({a}_{0},\Delta a\right)$ and $\mathcal{N}\left({\omega }_{0},\Delta \omega \right)$, respectively, with means ${a}_{0}$ and ${\omega }_{0}$, and standard deviations $\Delta a$ and $\Delta \omega$. The connectivity matrix ${\varvec{C}}$ was given by the HCP structural connectivity in a parcellation with $N$ = 1000 nodes (Schaefer parcellation). We numerically calculated the eigenvalues of the Jacobian matrix for different values of ${a}_{0}$ and the global coupling $g$ (normalized by the 2-norm of the connectivity matrix $\Vert C\Vert$) and we evaluated the stability of the origin. The origin is stable if ${\text{Re}}\left({\lambda }_{\text{max}}\right)<0$, where ${\lambda }_{\text{max}}$ is the eigenvalue with largest real part. Note logarithmic scale in the x-axis. *Grey*: the origin is unstable, i.e., ${\text{Re}}\left({\lambda }_{\text{max}}\right)>0$. *Blue*: the origin is stable, ${\text{Re}}\left({\lambda }_{\text{max}}\right)<0$, and ${a}_{j}<0$ for all nodes. *Light blue*: the origin is stable, ${\text{Re}}\left({\lambda }_{\text{max}}\right)<0$, and ${a}_{j}>0$ for at least one node. Parameters: $\Delta a=0.2$; $\Delta \omega =0.1\times 2\pi$. (**B**) Proportion of positive bifurcation parameters (${a}_{j}>0$), for $g/\Vert C\Vert =$ 0.7.

We studied the linear fluctuations $\delta {\varvec{z}}$ around the fixed point ${\varvec{z}}=0$, which is the solution of $\frac{d{\varvec{z}}}{dt}=0$ (Fig. 2A). In the linearized system the higher-order terms $\left( {\delta {\varvec{z}} \odot \delta \overline{\user2{z}}} \right)\delta {\varvec{z}}$ are discarded and only terms in the first-order in $\delta {\varvec{z}}$ are kept. Using the real and imaginary parts of the state variables, the evolution of the linear fluctuations $\delta {\varvec{u}}$ follows the stochastic linear equation:

10 $$\frac{d}{dt}\delta {\varvec{u}}={\varvec{A}}\delta {\varvec{u}}+{\varvec{\eta}},$$

where the 2*N*-dimensional column vector $\delta {\varvec{u}}=(\delta {\varvec{x}},\delta {\varvec{y}})={\left[{\delta x}_{1},\dots ,{\delta x}_{N},{\delta y}_{1},\dots ,{\delta y}_{N}\right]}^{T}$ contains the fluctuations of real and imaginary parts. The $2N\times 2N$ matrix ${\varvec{A}}$ is the Jacobian matrix of the system evaluated at the fixed point:

11 $${A}_{jk }={ \left.\frac{\partial {F}_{j}}{\partial {u}_{k}}\right|}_{0},$$

where ${F}_{j}=\left({a}_{j}-{{x}_{j}}^{2}-{{y}_{j}}^{2}\right){x}_{j}-{\omega }_{j}{y}_{j}+g\sum_{k=1}^{N}{C}_{jk}\left({x}_{k}-{x}_{j}\right)$ for $1\le j\le N$ (real parts), and ${F}_{j}=\left({a}_{j}-{{x}_{j}}^{2}-{{y}_{j}}^{2}\right){y}_{j}+{\omega }_{j}{x}_{j}+g\sum_{k=1}^{N}{C}_{jk}\left({y}_{k}-{y}_{j}\right)$ for $N+1\le j\le 2N$ (imaginary parts).

By evaluating the partial derivatives at the fixed point, the Jacobian matrix can be written as a block matrix:

12 $${\varvec{A}}=\left[\begin{array}{cc}{{\varvec{A}}}_{{\varvec{x}}{\varvec{x}}}& {{\varvec{A}}}_{{\varvec{x}}{\varvec{y}}}\\ {{\varvec{A}}}_{{\varvec{y}}{\varvec{x}}}& {{\varvec{A}}}_{{\varvec{y}}{\varvec{y}}}\end{array}\right],$$

where ${{\varvec{A}}}_{{\varvec{x}}{\varvec{x}}}$, ${{\varvec{A}}}_{{\varvec{x}}{\varvec{y}}}$, ${{\varvec{A}}}_{{\varvec{y}}{\varvec{x}}}$, ${{\varvec{A}}}_{{\varvec{y}}{\varvec{y}}}$ are $N\times N$ matrices given as: ${{\varvec{A}}}_{{\varvec{x}}{\varvec{x}}}={{\varvec{A}}}_{{\varvec{y}}{\varvec{y}}}\text{ = }{\text{diag}}\left({\varvec{a}}-g{\varvec{S}}\right)+g{\varvec{C}}$ and ${{\varvec{A}}}_{{\varvec{x}}{\varvec{y}}}={-{\varvec{A}}}_{{\varvec{y}}{\varvec{x}}}\text{ = }{\text{diag}}\left({\varvec{\omega}}\right)$, where $\text{diag(}{\varvec{v}}\text{)}$ is the diagonal matrix whose diagonal is the vector ${\varvec{v}}$. As shown below, the Jacobian matrix determines the statistics of the linear system. Note that the Jacobian depends on all the parameters of the model.

Given an initial condition $\delta {\varvec{u}}\left(0\right)$ at $t=0$, the general solution of a stochastic linear system such as Eq. (10) is given by^29^:

13 $$\delta {\varvec{u}}\left(t\right)={{\varvec{e}}}^{t{\varvec{A}}}\delta {\varvec{u}}\left(0\right)+\sigma {\int }_{0}^{t}{{\varvec{e}}}^{\left(t-s\right){\varvec{A}}}d{\varvec{W}}(s),$$

where ${\varvec{W}}$ is an 2N-dimensional Wiener process, $\sigma$ is the noise amplitude, and ${{\varvec{e}}}^{t{\varvec{A}}}$ is the exponential matrix defined as:

14 $${{\varvec{e}}}^{t{\varvec{A}}}=\sum_{k=0}^{\infty }\frac{1}{k!}{\left(t{\varvec{A}}\right)}^{k}={\varvec{I}}+t{\varvec{A}}+\frac{1}{2!}{\left(t{\varvec{A}}\right)}^{2}+\frac{1}{3!}{\left(t{\varvec{A}}\right)}^{3}+\cdots ,$$

where ${\varvec{I}}$ is the identity matrix. The right-hand side of Eq. (13) is the sum of the deterministic behavior plus a stochastic integral representing the diffusion due to noise.

The linearization is only valid if the origin ${\varvec{z}}=0$ is a stable solution of the system, i.e., if all eigenvalues of ${\varvec{A}}$ have negative real part. Note that, in complex representation, the Jacobian writes ${\varvec{A}}={\text{diag}}\left({\varvec{a}}+{\text{i}}{\varvec{\omega}}-g{\varvec{S}}\right)+g{\varvec{C}}={\text{diag}}\left({\varvec{a}}+{\text{i}}{\varvec{\omega}}\right)-g{\varvec{L}}$, where ${\varvec{L}}={\varvec{S}}-{\varvec{C}}$ is the Laplacian matrix of the network. It is known that the Laplacian matrix is positive semidefinite: the eigenvalues ${\mu }_{1}\le {\mu }_{2}\le \dots \le {\mu }_{N}$ of ${\varvec{L}}$ are real, nonnegative and ${\mu }_{1}=0$^30^. Let ${\lambda }_{j}$ be the eigenvalues of ${\varvec{A}}$, the origin is asymptotically stable if ${\text{Re}}\left({\lambda }_{\text{max}}\right)<0$, where ${\lambda }_{\text{max}}$ is the eigenvalue with largest real part. In the case of homogeneous local bifurcation and intrinsic frequency parameters, i.e., ${\text{diag}}\left({\varvec{a}}+{\text{i}}{\varvec{\omega}}\right)=\left(a+{\text{i}}\omega \right){\varvec{I}}$, the eigenvalues of ${\varvec{A}}$ relate to those of $-g{\varvec{L}}$ and we have ${\text{Re}}\left({\lambda }_{\text{max}}\right)=a-g{\mu }_{1}=a$. Thus, in this case, the origin is stable if $a<0$. For the heterogenous case, however, there is not a direct expression for ${\text{Re}}\left({\lambda }_{\text{max}}\right)$ which depends on the contribution of the matrices ${\text{diag}}\left({\varvec{a}}+{\text{i}}{\varvec{\omega}}\right)$ and $-g{\varvec{L}}$, and stability needs to be evaluated numerically. For the HCP coupling matrix and the heterogeneous case, we found that the stability of the origin fixed point increases as a function the global coupling $g$ and that, for sufficiently large $g$, the origin is stable even if ${a}_{j}>0$ for some nodes **(**Fig. 2A**)**. Indeed, for strong coupling and close to instability, the majority of nodes can have ${a}_{j}>0$ while the origin remains stable **(**Fig. 2B**)**. In other words, the focus solutions of single nodes can be unstable by themselves, but are stabilized by network interactions—as observed in simpler oscillator networks^31^.

#### Network statistics: covariances

In the following, we derive the network statistics of the linear system. The network mean activity (first order statistic) is trivial since fluctuations around the origin ${\varvec{z}}=0$ have null mean. A first interesting statistic is the covariance of the fluctuations around the origin, i.e., ${{\varvec{C}}}_{{\varvec{v}}}=\langle \delta {\varvec{u}}{\delta {\varvec{u}}}^{{\varvec{T}}}\rangle$, where the superscript $T$ denotes the transpose operator. For a stochastic linear system such as Eq. (10), the motion equation of the covariance matrix ${{\varvec{C}}}_{{\varvec{v}}}$ is given as:

15 $$\frac{d{{\varvec{C}}}_{{\varvec{v}}}}{dt}={\varvec{A}}{{\varvec{C}}}_{{\varvec{v}}}+{{\varvec{C}}}_{{\varvec{v}}}{{\varvec{A}}}^{{\varvec{T}}}+{{\varvec{Q}}}_{{\varvec{n}}},$$

where ${{\varvec{Q}}}_{{\varvec{n}}}=\langle {\varvec{\eta}}{{\varvec{\eta}}}^{{\varvec{T}}}\rangle$ is the covariance matrix of the noise. For uncorrelated noise, ${{\varvec{Q}}}_{{\varvec{n}}}$ is diagonal, i.e., ${{\varvec{Q}}}_{{\varvec{n}}}={\sigma }^{2}{\varvec{I}}$. The derivation of Eq. (15) is based on Eq. (10) which can be formally written as: $d\delta {\varvec{u}}={\varvec{A}}\delta {\varvec{u}}dt+d{\varvec{W}}$, where ${\varvec{W}}$ is an 2*N*-dimensional Wiener process with covariance $\langle d{\varvec{W}}{d{\varvec{W}}}^{{\varvec{T}}}\rangle ={{\varvec{Q}}}_{{\varvec{n}}}dt$. Using Itô’s stochastic calculus, we get $d\left(\delta {\varvec{u}}{\delta {\varvec{u}}}^{{\varvec{T}}}\right)=d\left(\delta {\varvec{u}}\right){\delta {\varvec{u}}}^{{\varvec{T}}}+\delta {\varvec{u}}d\left({\delta {\varvec{u}}}^{{\varvec{T}}}\right)+d\left(\delta {\varvec{u}}\right)d\left({\delta {\varvec{u}}}^{{\varvec{T}}}\right)$, and thus: $d\left(\delta {\varvec{u}}{\delta {\varvec{u}}}^{{\varvec{T}}}\right)=\left({\varvec{A}}\delta {\varvec{u}}dt+d{\varvec{W}}\right){\delta {\varvec{u}}}^{{\varvec{T}}}+\delta {\varvec{u}}\left({{\delta {\varvec{u}}}^{{\varvec{T}}}{\varvec{A}}}^{{\varvec{T}}}dt+d{{\varvec{W}}}^{{\varvec{T}}}\right)+\left({\varvec{A}}\delta {\varvec{u}}dt+d{\varvec{W}}\right)\left({{\delta {\varvec{u}}}^{{\varvec{T}}}{\varvec{A}}}^{{\varvec{T}}}dt+d{{\varvec{W}}}^{{\varvec{T}}}\right)$. This allows to calculate the evolution of the covariance $d\langle \delta {\varvec{u}}{\delta {\varvec{u}}}^{{\varvec{T}}}\rangle$. Since $\langle \delta {\varvec{u}}{d{\varvec{W}}}^{{\varvec{T}}}\rangle =0$, taking the expectations and keeping terms in first order of the differential $dt$ (since ${dt}^{2}$ can be made arbitrarily small), we obtain: $d\langle \delta {\varvec{u}}{\delta {\varvec{u}}}^{{\varvec{T}}}\rangle ={\varvec{A}}\langle \delta {\varvec{u}}{\delta {\varvec{u}}}^{{\varvec{T}}}\rangle dt+\langle \delta {\varvec{u}}{\delta {\varvec{u}}}^{{\varvec{T}}}\rangle {{\varvec{A}}}^{{\varvec{T}}}dt+{{\varvec{Q}}}_{{\varvec{n}}}dt$.

The stationary covariance matrix can be obtained by solving $\frac{d{{\varvec{C}}}_{{\varvec{v}}}}{dt}=0$, which leads to the following algebraic equation:

16 $${\varvec{A}}{{\varvec{C}}}_{{\varvec{v}}}+{{\varvec{C}}}_{{\varvec{v}}}{{\varvec{A}}}^{{\varvec{T}}}+{{\varvec{Q}}}_{{\varvec{n}}}=0,$$

Equation (16) is an algebraic Lyapunov equation that has a unique solution provided that ${\varvec{A}}$ is asymptotically stable. The Lyapunov equation can be solved using the eigen-decomposition of the Jacobian matrix. Let ${\varvec{A}}\boldsymbol{ }=\boldsymbol{ }{\varvec{V}}{\varvec{D}}{{\varvec{V}}}^{-1}$, where ${\varvec{D}}$ is a diagonal matrix containing the eigenvalues of ${\varvec{A}}$, denoted ${\lambda }_{i}$, and the columns of matrix ${\varvec{V}}$ are the eigenvectors of ${\varvec{A}}$. Multiplying Eq. (16) by ${{\varvec{V}}}^{-1}$ from the left and by the conjugate transpose of ${{\varvec{V}}}^{-1}$, noted ${\varvec{V}}^{ - \dag }$, from the right we get:

17 $${\varvec{C}}_{{\varvec{v}}} = {\varvec{VMV}}^{ - \dag } ,$$

where the matrix ${\varvec{M}}$ is given as: ${M}_{ij}={-\widetilde{Q}}_{ij}/({\lambda }_{i}+{\lambda }_{i}^{*})$ and $\tilde{\user2{Q}} = {\varvec{V}}^{ - 1} {\varvec{Q}}_{{\varvec{n}}} {\varvec{V}}^{ - \dag }$. A fast, stable numerical solution of Eq. (16) can be obtained using the MatLab function *lyap.m* that uses the Bartels-Stewart method^32^ based on the Schur decomposition of the matrix ${\varvec{A}}$.

Moreover, knowledge of the Jacobian matrix and the stationary covariance gives the stationary lagged covariances of the state variables, defined as ${{\varvec{C}}}_{{\varvec{v}}}(\tau )=\langle \delta {\varvec{u}}{(t+\tau )\delta {\varvec{u}}(t)}^{{\varvec{T}}}\rangle$. Using the general solution of the system given by Eq. (13), we get:

18 $${{\varvec{C}}}_{{\varvec{v}}}\left(\tau \right)={{\varvec{e}}}^{\tau {\varvec{A}}}\langle \delta {\varvec{u}}{(t)\delta {\varvec{u}}(t)}^{{\varvec{T}}}\rangle ={{\varvec{e}}}^{\tau {\varvec{A}}}{{\varvec{C}}}_{{\varvec{v}}}\left(0\right),$$

where ${{\varvec{C}}}_{{\varvec{v}}}\left(0\right)={{\varvec{C}}}_{{\varvec{v}}}$ is the covariance matrix (i.e., zero-lag). The lagged covariance has been used to described the temporal structure of whole-brain activity^33^.

#### Network statistics: power spectral densities

In the frequency domain, the power spectral density (PSD) of fluctuations around the fixed point is also determined by the Jacobian matrix. Taking the Fourier transform $\mathcal{F}$ of Eq. (10), we get:

19 $$\mathcal{F}\left[\frac{d\delta {\varvec{u}}}{dt}\right]={\varvec{A}}\mathcal{F}\left[\delta {\varvec{u}}\right]+\mathcal{F}\left[{\varvec{\eta}}\right],$$

20 $$-{\text{i}}2\pi \nu{\varvec{\delta}}\widetilde{{\varvec{u}}}\left(\nu \right)={\varvec{A}}{\varvec{\delta}}\widetilde{{\varvec{u}}}\left(\nu \right)+\widetilde{{\varvec{\eta}}}\left(\nu \right),$$

where ${\varvec{\delta}}\widetilde{{\varvec{u}}}\left(\nu \right)$ and $\widetilde{{\varvec{\eta}}}\left(\nu \right)$ are the Fourier transforms of $\delta {\varvec{u}}\left(t\right)$ and ${\varvec{\eta}}\left(t\right)$ at frequency $\nu$, respectively. Using the relation ${\varvec{\delta}}\widetilde{{\varvec{u}}}=-{\left({\varvec{A}}+{\text{i}}2\pi \nu {\varvec{I}}\right)}^{-1}\widetilde{{\varvec{\eta}}}$, we get the cross-spectrum of the linear fluctuations:

21 $${\varvec{\psi}}\left( \nu \right) = \langle \user2{\delta \tilde{u}\delta \tilde{u}}^{\dag } \rangle = \left( {{\varvec{A}} + {\text{i}}2\pi \nu {\varvec{I}}} \right)^{ - 1} {\varvec{Q}}_{{\varvec{n}}} \left( {{\varvec{A}}^{{\varvec{T}}} - {\text{i}}2\pi \nu {\varvec{I}}} \right)^{ - 1} .$$

The real part of the cross-spectrum (also called co-spectrum) represents the simultaneous covariance at frequency $\nu$. Its imaginary part (called quadrature spectrum) is the covariance of time-series lagged by a phase $\pi /2$ at frequency $\nu$. At each frequency $\nu$, the PSDs of the nodes, ${\phi }_{j}\left(\nu \right)$, are given by the diagonal terms of ${\varvec{\psi}}\left(\nu \right)$ and the coherence between nodes, ${\gamma }_{jk}\left(\nu \right)$, is given by the normalized cross-spectrum, i.e., ${\gamma }_{jk}\left(\nu \right)={\psi }_{jk}\left(\nu \right)/\sqrt{{\phi }_{j}\left(\nu \right){\phi }_{k}\left(\nu \right)}$^34^. For uncorrelated noise, the PSD is given as:

22 $${\phi }_{j}\left(\nu \right)={\psi }_{jj}\left(\nu \right)={\sigma }^{2}\sum_{k}{\left|{J}_{jk}\right|}^{2},$$

where $${{\varvec{J}}=\left({\varvec{A}}+{\text{i}}2\pi \nu {\varvec{I}}\right)}^{-1}$$.

The Fourier transform is also a useful tool to study the system in the case of time-delays. Consider the nonlinear Hopf network with delayed interactions:

23 $${\dot{z}}_{j}(t)=\left({a}_{j}+{\text{i}}{\omega }_{j}\right){z}_{j}(t)-{\left|{z}_{j}\left(t\right)\right|}^{2}{z}_{j}(t)+g\sum_{k=1}^{N}{C}_{jk}\left[{z}_{k}\left(t-{\tau }_{jk}\right)-{z}_{j}\left(t\right)\right]+{\eta }_{j}(t),$$

where ${\tau }_{jk}$ represents the time-delay of the interaction between nodes $j$ and $k$. For simplicity, one can assume that ${\tau }_{jk}$ is given by the Euclidean distance between nodes $j$ and $k$ divided by a constant transmission velocity $v$. Delayed-interactions can be treated in the Fourier space, since the change of variable ${t}^{\prime}=t-{\tau }_{jk}$ leads to $\mathcal{F}\left[{z}_{k}\left(t-{\tau }_{jk}\right)\right]={\int }_{-\infty }^{+\infty }{z}_{k}\left(t-{\tau }_{jk}\right){e}^{-2\pi \nu t}dt={e}^{2\pi \nu {\tau }_{jk}}{\int }_{-\infty }^{+\infty }{z}_{k}\left(t^{\prime}\right){e}^{-2\pi \nu {t}^{\prime}}dt^{\prime}={e}^{2\pi \nu {\tau }_{jk}}\mathcal{F}\left[{z}_{k}\left(t\right)\right]$. Using the linear approximation and the Fourier transform, we get:

24 $${\mathcal{F}}\left[ {\frac{{d\delta {\varvec{u}}}}{dt}} \right] = {\varvec{B}}{\mathcal{F}}\left[ {\delta {\varvec{u}}} \right] + g\left( {{\varvec{C}} \odot e^{{{\text{i}}2\pi \nu {{\varvec{\Gamma}}}}} } \right){\mathcal{F}}\left[ {\delta {\varvec{u}}} \right] + {\mathcal{F}}\left[ {\varvec{\eta}} \right],$$

25 $$- {\text{i}}2\pi \nu \user2{\delta \tilde{u}}\left( \nu \right) = \user2{B\delta \tilde{u}}\left( \nu \right) + g\left( {{\varvec{C}} \odot e^{{{\text{i}}2\pi \nu {{\varvec{\Gamma}}}}} } \right)\user2{\delta \tilde{u}}\left( \nu \right) + \tilde{\user2{\eta }}\left( \nu \right),$$

where ${\varvec{\Gamma}}$ is the matrix containing the delays, i.e., ${\Gamma }_{jk}={\tau }_{jk}$, the elements of ${\varvec{C}} \odot e^{{{\text{i}}2\pi {{\varvec{\Gamma}}}}}$ are ${C}_{jk}{e}^{{\text{i}}2\pi {\tau }_{jk}}$, and ${\varvec{B}}$ is the block matrix given by:

26 $${\varvec{B}}=\left[\begin{array}{cc}{{\varvec{B}}}_{{\varvec{x}}{\varvec{x}}}& {{\varvec{B}}}_{{\varvec{x}}{\varvec{y}}}\\ {{\varvec{B}}}_{{\varvec{y}}{\varvec{x}}}& {{\varvec{B}}}_{{\varvec{y}}{\varvec{y}}}\end{array}\right],$$

where ${{\varvec{B}}}_{{\varvec{x}}{\varvec{x}}}={{\varvec{B}}}_{{\varvec{y}}{\varvec{y}}}\text{ = }{\text{diag}}\left({\varvec{a}}-g{\varvec{S}}\right)$ and ${{\varvec{B}}}_{{\varvec{x}}{\varvec{y}}}={-{\varvec{B}}}_{{\varvec{y}}{\varvec{x}}}\text{ = }{\text{diag}}\left({\varvec{\omega}}\right)$. From Eq. (25), we get ${\varvec{\delta}}\widetilde{{\varvec{u}}}=-\left( {{\varvec{B}} + g{\varvec{C}} \odot e^{{{\text{i}}2\pi {{\varvec{\Gamma}}}}} + {\text{i}}2\pi \nu {\varvec{I}}} \right)^{ - 1}\tilde{\user2{\eta }}$, and thus the cross-spectrum is given by:

27 $${\varvec{\psi}}\left( \nu \right) = \langle \user2{\delta \tilde{u}\delta \tilde{u}}^{\dag } \rangle = {\varvec{UQ}}_{{\varvec{n}}} {\varvec{U}}^{\dag } ,$$

with ${\varvec{U}} = \left( {{\varvec{B}} + g{\varvec{C}} \odot e^{{{\text{i}}2\pi {{\varvec{\Gamma}}}}} + {\text{i}}2\pi \nu {\varvec{I}}} \right)^{ - 1}$. From the cross-spectrum ${\varvec{\psi}}$ we can obtain the PSD of each node (i.e., diagonal terms), the lagged-covariances (i.e., the inverse Fourier transform of the cross-spectrum), and the covariance matrix ${{\varvec{C}}}_{{\varvec{v}}}$ by integrating the real part of ${\varvec{\psi}}$ over frequencies:

28 $${{\varvec{C}}}_{{\varvec{v}}}=2{\int }_{0}^{\infty }{\text{Re}}\left[{\varvec{\psi}}\left(\nu \right)\right]d\nu .$$

In summary, in the linear approximation, the stationary instantaneous and lagged covariance matrices, the cross-spectrum, and the PSDs of the model can be obtained through algebraic operations including the Jacobian matrix. This can be done both in the homogeneous and the heterogeneous cases, and also in the presence of time delays.

### Comparison with stochastic simulations

We compared the predictions of the linear approximation against the statistics obtained using stochastic simulations of the nonlinear model. The coupling matrix was given by the human dMRI connectome from HCP, with $N=$ 1000 nodes. The model parameters ${\varvec{a}}$ and ${\varvec{\omega}}$ were drawn from normal distributions $\mathcal{N}\left({a}_{0},\Delta a\right)$ and $\mathcal{N}\left({\omega }_{0},\Delta \omega \right)$, respectively, with means ${a}_{0}$ and ${\omega }_{0}$, and standard deviations $\Delta a$ and $\Delta \omega$. We simulated the system for $T=$ 3 min after letting it reach the stationary regime and we used $n=$ 100 realizations of the system with different random initial conditions.

**Figure 3:**
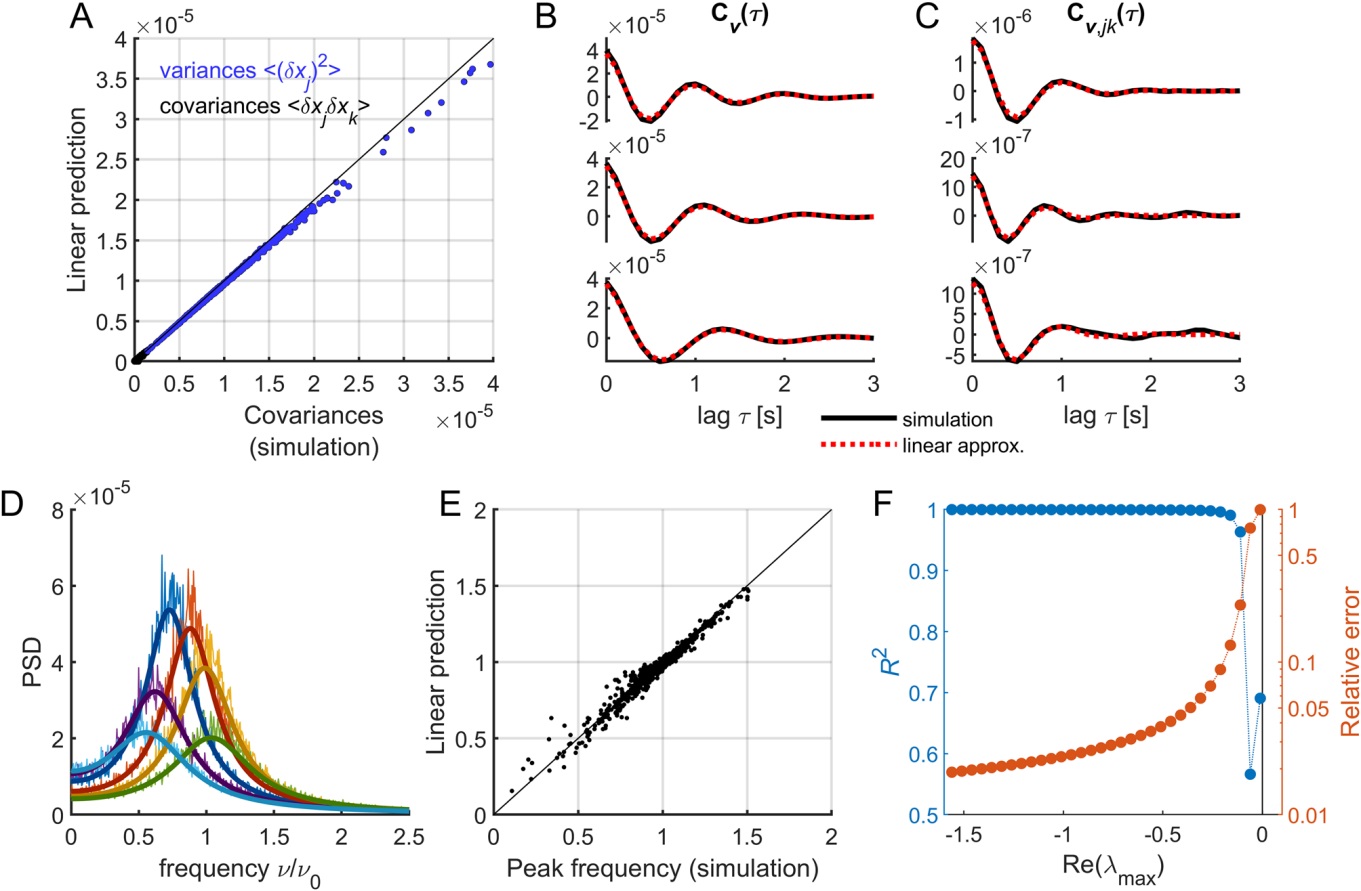
Comparison with numerical simulations. (**A**) Comparison between variances and covariances obtained using numerical simulations and the linear approximation. The black line indicates the identity line. (**B**,**C**) Autocovariances (**B**) and lagged covariances (**C**) for numerical simulations (black trace) and the linear approximation (red dotted trace) for three example nodes (**B**) and pairs of nodes (**C**). (**D**) PSD for six example nodes and their linear predictions (solid lines). The frequency was normalized by the average intrinsic frequency ${\nu }_{0}={\omega }_{0}/\left(2\pi \right)$. (**E**) Comparison between the peak frequencies (normalized by ${\nu }_{0}$) obtained using numerical simulations and the linear approximation. The black line indicates the identity line. Model parameters for panels (**A**–**E**): ${a}_{0}=-1$; $\Delta a=0.3$; $g=3$; ${\omega }_{0}=2\pi$; $\Delta \omega =0.2\times 2\pi$; $\sigma =0.01$. (**F**) Accuracy of the prediction for different values of ${\text{Re}}\left({\lambda }_{\text{max}}\right)$. The origin is stable for ${\text{Re}}\left({\lambda }_{\text{max}}\right)<0$. We quantified the goodness of the prediction through the R-squared value (${R}^{2}$) of the correlation between covariances obtained from numerical simulations and those obtained with the linear approximation. In the analysis presented in panel (F) we used a subsample of the network, i.e., $N$ = 250 nodes. Model parameters: $\Delta a=0.3$; $g=3$; ${\omega }_{0}=2\pi$; $\Delta \omega =0.2\times 2\pi$; $\sigma =0.001$.

We used the linear approximation to study the fluctuations around the origin. We first examined the predictions of the linear approximation when the stability of the origin is strong (${\text{Re}}\left({\lambda }_{\text{max}}\right)<-1$). In this case, the approximation accurately estimates the covariances (Fig. 3A), the auto- and cross-covariances (Fig. 3B,C), and the PSDs (Fig. 3D,E). To study the accuracy of the prediction as a function of the origin’s stability, we varied the local bifurcation parameter ${a}_{0}$ in the homogeneous case (i.e., $\Delta a=0$). This analysis, that requires to simulate the system for different parameters ${a}_{0}$, was done using a subsampled of the network, with $N$ = 250 nodes (see Methods). As the origin loses stability, nonlinear terms become non-negligible, it is thus expected that the linear approximation fails close to ${\text{Re}}\left({\lambda }_{\text{max}}\right)\to 0$. We quantified the goodness of the prediction through two measures: (i) the R-squared value (${R}^{2}$) of the correlation between covariances obtained from numerical simulations ${{\varvec{C}}}_{{\varvec{v}}}^{\text{sim}}$ and those obtained with the linear approximation ${{\varvec{C}}}_{{\varvec{v}}}^{\text{lin}}$, and *ii*) the relative error ($E$) between the matrices using the Frobenius norm: $E=\Vert {{\varvec{C}}}_{{\varvec{v}}}^{\text{sim}}\Vert \Vert {{\varvec{C}}}_{{\varvec{v}}}^{\text{lin}}\Vert /\Vert {{\varvec{C}}}_{{\varvec{v}}}^{\text{sim}}\Vert$ (Fig. 3F). We found that the linear approximation accurately estimates the covariances (${R}^{2}>.99$ and $E<0.1$) for ${\text{Re}}\left({\lambda }_{\text{max}}\right)<-0.15$.

**Figure 4:**
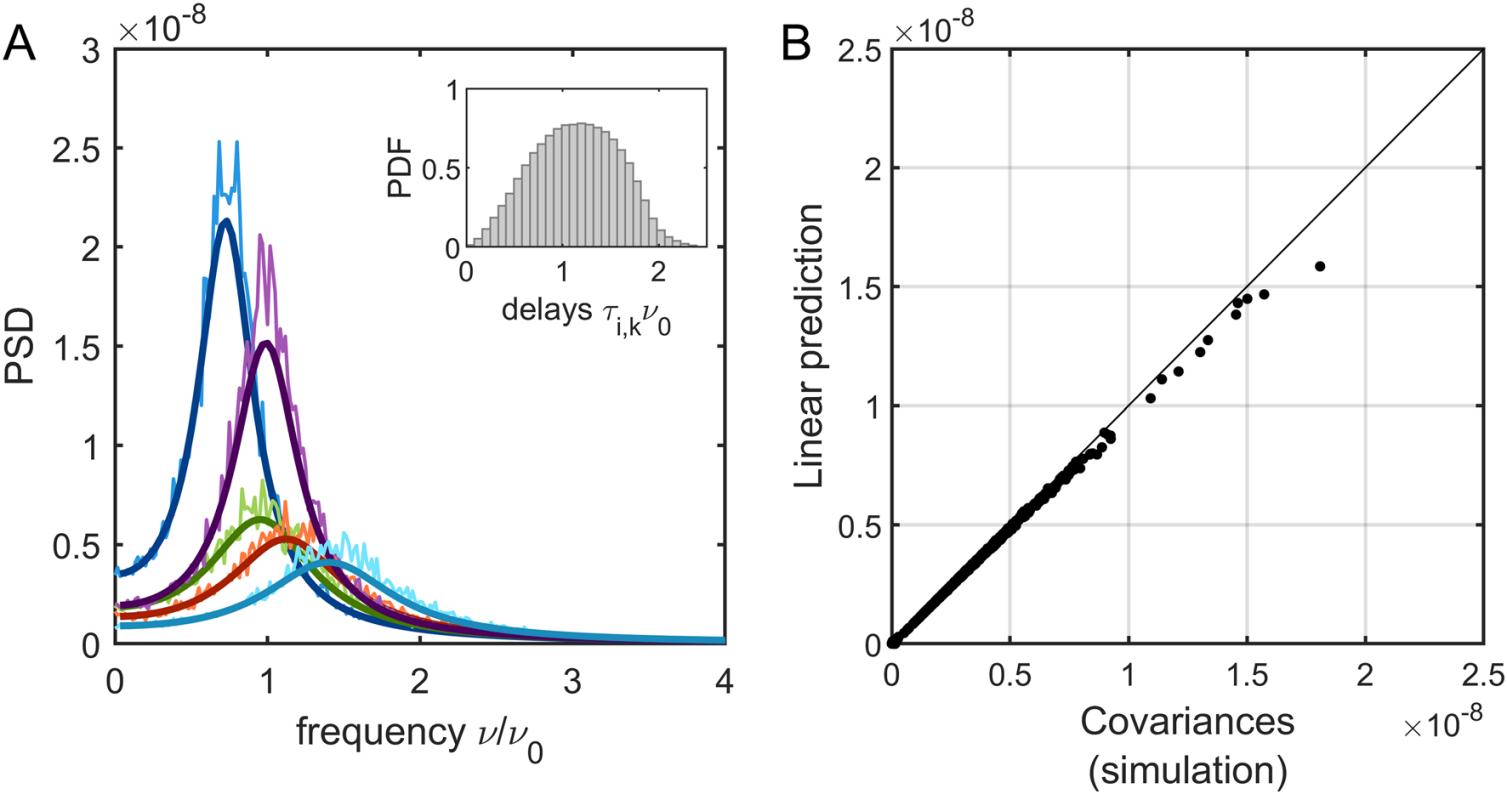
Delay-coupled system. (**A**) PSD for five example nodes and their linear predictions (solid lines). The frequency was normalized by the average intrinsic frequency ${\nu }_{0}={\omega }_{0}/\left(2\pi \right)$. The transmission velocity was $v$ = 0.07 m/s. *Inset*: distribution of time delays (normalized by ${\nu }_{0}$). (**B**) Comparison between variances and covariances obtained using numerical simulations and the linear approximation. The black line indicates the identity line. Model parameters: ${a}_{0}=-1$; $\Delta a=0.3$; $g=3$; ${\omega }_{0}=2\pi$; $\Delta \omega =0.2\times 2\pi$; $\sigma =0.0002$.

We also evaluated the predictions of the linear approximation in the case of time-delays. The delay-coupled Hopf model has been recently studied using numerical simulations^35^. In this case, the interaction delays between nodes can be approximated using the Euclidean distance between brain regions divided by a transmission velocity $v$. Here, we used the distances from the HCP data, which yield an average distance between nodes equal to 79 mm. The intrinsic frequencies were chosen from a normal distribution centered on $\frac{{\omega }_{0}}{2\pi }={\nu }_{0}=1$ Hz and with standard deviation equal to $\frac{\Delta \omega }{2\pi }=$ 0.2 Hz. For this example, we chose a transmission velocity $v$ such that the average transmission delay $D$ is of the same order of the average intrinsic period of the network, i.e., $D\sim {{\nu }_{0}}^{-1}$. The parameters ${\varvec{a}}$ were drawn from the normal distribution $\mathcal{N}\left({a}_{0},\Delta a\right)$, with ${a}_{0}=-1$ and $\Delta a=$ 0.3. As previously, we simulated the system for $T=$ 3 min after letting it reach the stationary regime and we used $n=100$ realizations of the system with different random initial conditions. The linear approximation accurately approximates the PSD of the nodes (Fig. 4A). Moreover, integration of the cross-spectrum, obtained using the linear approximation, gives an accurate prediction of covariances (Fig. 4B).

### Parameter exploration and data fitting

**Figure 5:**
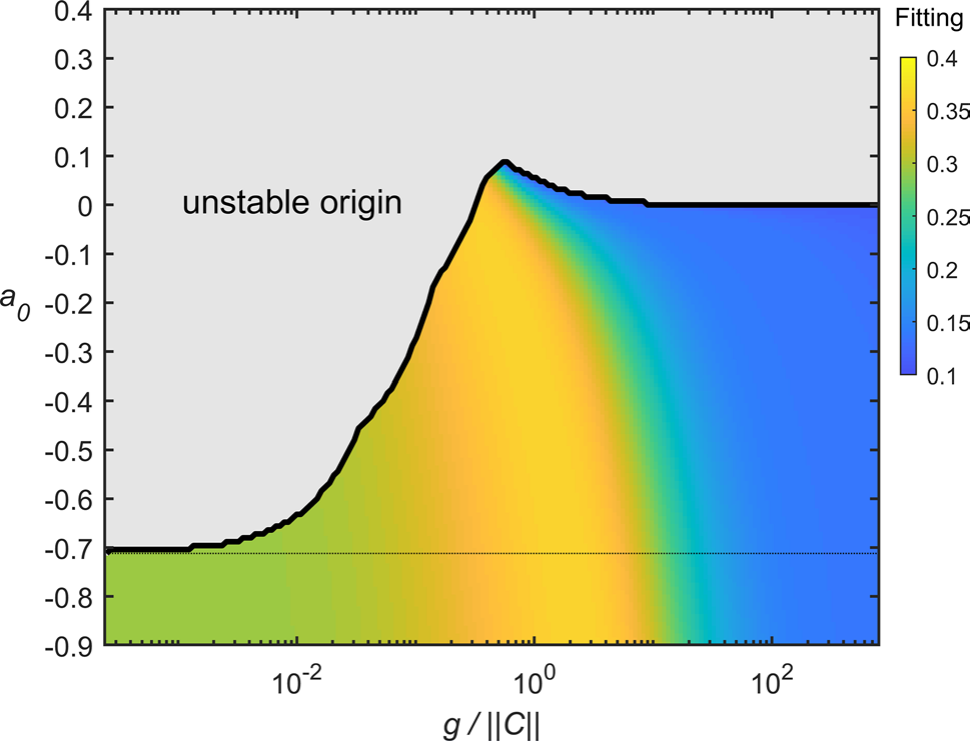
FC prediction in parameter space. Correlation between FC matrices obtain from the data and the linearized Hopf model, for varying mean local bifurcation parameter and global coupling. *Grey*: the origin is unstable, i.e., ${\text{Re}}\left({\lambda }_{\text{max}}\right)>0$. Between the horizontal line and the grey zone, the nodes can have ${a}_{j}>0$ while the origin remains stable. Note the logarithmic scale of the x-axis.

Finally, we studied how well the linear approximation predicts the correlations of resting-state (rs-) fMRI signals. For this, we analyzed rs-fMRI signals from the HCP, from 1003 participants. First, we calculated the correlation matrix (or functional connectivity, FC) averaged over participants, in the parcellation with $N=$ 1000 nodes. Second, we computed the FC for the heterogenous linearized Hopf model constraint by the HCP dMRI connectivity matrix. Finally, we compute the correlation between FC matrices obtain from the data and from the linearized Hopf model. The model parameters ${\varvec{a}}$ and ${\varvec{\omega}}$ were drawn from normal distributions $\mathcal{N}\left({a}_{0},\Delta a\right)$ and $\mathcal{N}\left({\omega }_{0},\Delta \omega \right)$, respectively, with $\Delta a=0.2$, ${\omega }_{0}=2\pi$, and $\Delta \omega =0.1$. Note that, here, the local parameters ${a}_{j}$ and ${\omega }_{j}$ were taken from normal distributions and were not fitted/optimized using the data as in previous work^20^. We evaluated the fitting of the empirical FC in the parameter space $\left({a}_{0},g\right)$, for varying mean local bifurcation parameter and global coupling (Fig. 5). We found that, for this particular example, the best fit of the FC was obtained when the coupling was high enough with respect to the norm of the connectivity matrix (i.e., $\frac{g}{\Vert C\Vert }\sim$ 1–100). The fitting values are similar to what was found with previous numerical simulations with the same parcellation^36^. In that previous work, however, long-range connections were added to the connectivity, which improve the fit. Also, in previous studies^15,16^, a narrow band-pass filter was applied to the fMRI signals, thus making the signals strongly oscillatory, which might explain the fit increase close to the onset of self-sustained oscillations.

## Discussion

Using a linear approximation, we have derived network statistics of the Hopf whole-brain model. The linearization allows analytical estimation of the stationary instantaneous and lagged covariance matrices, the cross-spectrum, and the PSDs of the model. This can be done in the most general form of the model, namely in the delay-coupled heterogeneous case. The linearization provides good estimates of these quantities as soon as non-linear terms do not dominate (as it is the case sufficiently close or beyond the bifurcation). This occurs when the origin is stable. Exploration of the parameter space, for which the origin destabilizes and dynamics are strongly nonlinear, could be treated using approximations more sophisticated than the linear approximation, for example, using higher-order phase reduction^37^.

Synchronization among brain regions has been studied in multiple previous studies using different neuroimaging techniques^10,18, 38–42^. The present model is a canonical model to describe, at a phenomenological level, the synchronization of oscillators with phase and amplitude interactions, previously used to study large-scale brain dynamics^15–18, 20, 22^. However, the neuronal/synaptic mechanisms underlying the brain’s large-scale synchronization are not fully understood. Noisy oscillations around a fixed point can be understood using more realistic, yet still simple, models composed of interconnected excitatory and inhibitory neural populations such as the Wilson-Cowan model^43^ or the stabilized supralinear network^44^, for which linear fluctuations can be studied in light of the biological interpretation of the different parameters. It is worth noting that the linear fluctuations around the fixed point are rich in structure, as shown here by their structured covariance and cross-spectrum which are determined by local dynamics, network interactions, network stability, time-delays, and noise propagation. Even richer dynamics could emerge in the case of strongly nonlinear dynamics, which might be the subject of future research.

There are several applications of the present framework. The estimated network statistics can be used to track changes in the brain state, e.g., in the case of low-level states of consciousness^17–21^, anesthesia^20,45^, sleep^16^, etc., or to evaluate the effect of lesions in the connectome^46–49^.

We here tested the model predictions using rs-fMRI data, but the model can be used to approximate MEG data in different frequency bands^28,35^. The slow time scale of fMRI signals allows to neglect the effect of conduction delays between the different brain regions, which are orders of magnitude faster—tens of milliseconds^50^—than the periods of the model oscillators, and treat the interactions as instantaneous. In the case of MEG data, however, delayed interactions can have an important effect for sufficiently fast frequency bands. Thus, the linear approximation of delay-coupled Hopf whole-brain model derived here can represent a valuable tool to study the PSDs and cross-spectrum of MEG, which are well-established methods for FC analysis in the frequency domain^51–54^.

Furthermore, recent studies suggest that dynamics out of equilibrium are relevant to describe the whole-brain^55–57^. The present model can be used to track non-stationarities by assuming that changes in parameters are sufficiently slow relative to the time it takes for the system to reach equilibrium^58^. In this way, using the linear model to fit the stationary statistics of the system measured in short time windows, it is possible to infer the change in network parameters over time.

Finally, since the goal of the present study was to derive the linear statistics of the model, rather than fitting functional data, we used models for which the local parameters were not estimated from the data, opposite to previous studies^15,20^. Future research could combine the present linear approximation with algorithms to optimize the parameters of the model, such as the $N$ local bifurcation parameters. This can be achieved using genetic algorithms applied to infer optimal local parameters^59^, allowing to compare the learned parameters in different brain states, disorders, or across aging, for example. The use of the linear approximation would allow to estimate the parameters for large *N*.

In all the above applications, one would need to systematically verify that the origin of the model is a stable fixed point and that the real part of the leading eigenvalue does not approach zero.

## Methods

### Neuroimaging ethics

The Washington University–University of Minnesota (WU-Minn HCP) Consortium obtained full written informed consent from all participants to study procedures and data sharing outlined by HCP, and research procedures and ethical guidelines were followed in accordance with Washington University institutional review board approval.

### Functional MRI data

In this study we analyzed publicly available rs-fMRI data from the Human Connectome Project (HCP), from 1003 participants. The participants were scanned on a 3 T connectome-Skyra scanner (Siemens). The rs-fMRI data was acquired for approximately 15 min, with eyes open and relaxed fixation on a projected bright cross-hair on a dark background. The HCP website (https://www.humanconnectome.org/) provides the details of participants, the acquisition protocol and preprocessing of the functional data.

### Parcellation

Schaefer and colleagues created a publicly available population atlas of cerebral cortical parcellation based on estimation from a large data set (*n* = 1489)^60^. They provide parcellations of regions of interest (ROIs) available in surface spaces, as well as MNI152 volumetric space. We used the Schaefer parcellation with 1000 areas and estimated the Euclidean distances from the MNI152 volumetric space^60^ and extracted the timeseries from HCP using the surface space version. Finally, for the analysis presented in Fig. 3F, we subsampled the connectivity by choosing only 250 ROIs. This allowed us to simulate the stochastic nonlinear dynamical system for a large amount of repetitions, initial conditions, and varying parameters.

### Structural connectivity using dMRI

Structural connectivity was estimated from diffusion spectrum and T2-weighted imaging data from 32 participants from the HCP database, scanned over 89 min. Acquisition parameters are described in detail in the HCP website^61^. The freely available Lead-DBS software package (http://www.lead-dbs.org/) provided the preprocessing which is described in detail in Horn and colleagues^62^. Standardized methods in Lead-DBS were used to produce the structural connectomes for the Schaefer parcellation scheme^60^. The connectivity weight ${C}_{ij}={C}_{ji}$ was given by the number of fibers connecting two brain regions. To have values between 0 and 1, we normalized the weights by dividing them by the largest value, i.e., ${\text{max}}({\varvec{C}})$.

### Statistics and reproducibility

The goodness of the linear prediction of fMRI FC was given by the Pearson correlation between the vectorized FC averaged over all subjects and the model FC for all combinations of parameters $\left({a}_{0},g\right)$ (Fig. 5).

Stochastic numerical simulations were performed using Euler’s method, with a simulation step size equal to 0.001 s and 0.005 s in the absence and presence of delays, respectively. The system was simulated for $T=$ 3 min after letting it reach the stationary regime after 20 s; the stochastic simulations were repeated $n$ times with different random initial conditions ($n$ = 100). For the subsampled system of 250 nodes (Fig. 3F) we used: $T=$ 10 min and $n$ = 200. The PSDs of simulated time-series were estimated using the fast Fourier transform.

MATLAB (R2021a) software was used to perform all analyses and to simulated the model. Numerical simulations were performed in a 50-nodes computer cluster (Intel® Xeon® E5-2684 at 2.1 Ghz, 256 GB RAM, 1 TB disk).

## Data Availability

We used a publicly available dataset of dMRI and fMRI data from the Human Connectome Project (HCP). Structural connectivity was estimated from diffusion spectrum and T2-weighted imaging data from 32 participants from the HCP database. fMRI data was acquired from 1003 participants. The HCP dataset is available at https://www.humanconnectome.org/study/hcp-young-adult.

## Code availability

The codes to perform the numerical simulations and to estimate the network statistics using the linear approximation are available at https://github.com/adrianponce/Linear-Hopf-model. In this repository, we also included the structural and functional connectivity matrices used here. The provided codes and matrices are sufficient to replicate the present study.
